# A cross-sectional analysis of occupational stress and mental health among migrant healthcare workers in Ireland

**DOI:** 10.1016/j.jmh.2025.100325

**Published:** 2025-03-25

**Authors:** Sindhu Thankachen, Zubair Kabir, Anvar Sadath

**Affiliations:** a Clinical Nurse Manager 2, Occupational Health, Mater Private Hospital, Dublin, Ireland; b School of Public Health, University College Cork, Ireland; c National Suicide Research Foundation, University College Cork, Ireland; d Grace Abbott School of Social Work, College of Public Affairs and Community Service, University of Nebraska at Omaha, United States

**Keywords:** Healthcare workers, Migrant nurses, Migrant healthcare assistants, Occupational stress, Psychological distress, Mental health

## Abstract

**Background:**

The migration of healthcare workers (HCWs) to European countries has witnessed a recent increase in both number and diversity. While facing various socio-cultural challenges in the host country, it becomes imperative to investigate the mental health of these workers, given the significance for their well-being and the safety of patients. This study aims to assess the extent of occupational stress and psychological distress among migrant healthcare workers in Ireland, along with exploring the contributing factors.

**Methods:**

A cross-sectional online survey, utilizing Google Forms, was conducted via social media platforms among overseas nursing and Healthcare Assistants currently employed in the Republic of Ireland. Occupational stress and psychological distress were assessed using the Brief Job Stress Questionnaire (BJSQ) and the Kessler Psychological Distress Scale (K6). Multivariable linear regression models were employed to identify significant predictors of occupational stress and psychological distress.

**Results:**

The response rate was 35.8 (*n* = 447) from 1250 invitees. The majority of participants were nurses (93 %), largely from India (72.7 %), followed by the Philippines (17.7 %). The healthcare workers reported moderate to high job stress, with elevated scores in quantitative overloads, mental demands at work, and physical work demands. Migrant healthcare workers with <10 years of work experience in Ireland perceived higher job stress (*t* = 2.826; β = 0.154; *p* = 0.005) and psychological distress (*t* = 5.666; β = 0.303; *p* = 0.000). Working in private healthcare sectors was associated with a lower perception of job stress (*t* = -3.077; β = 0.15; *p* = 0.002) and psychological distress (*t* = -2.643; β = 0.126; *p* = 0.009).

**Conclusion:**

The findings highlight that HCWs experience moderate to high job stress, particularly those with <10 years of experience. Psychological distress was also prevalent, with private-sector employment associated with lower stress levels. These results emphasize the need for targeted interventions to support migrant HCWs’ well-being.

## Background

In recent decades, there has been an increase in the international migration of Healthcare Workers (HCWs), largely driven by transnationalization of governance and policy responses (Yeates and Pillinger, 2018). HCWs from developing countries primarily relocate to developed countries for several reasons, namely, an efficient healthcare system, a good working environment, financial and job security, improved working conditions, and career progression (Toyin-Thomas et al., 2023). There has been a global shortage of HCW workforce, particularly nurses and midwives, who constitute >50 % of the current deficit in HCW workforce (World Health Organization, 2022). The number of migrant nurses and doctors has increased by 60 % within the Organisation for Economic Co-operation and Development (OECD) since 2004 (ILO, 2015). India and the Philippines are sourcing the largest international nursing workforce (ILO, 2015; Schilgen et al., 2017).

HCWs' migration to European countries has increased in number and diversity in recent years (Villarroel et al., 2019). Ireland, historically known for its emigration, is currently experiencing high immigration, and its healthcare system highly depends on internationally trained nurses. In 2022, a total of 331,100 individuals were part of the Irish human health and social work sector (Central Statistics Office (CSO), 2022). The Nursing and Midwifery Board of Ireland (NMBI) recorded 89,496 registered nurses and midwives, with 84,213 actively engaged in their profession (NMBI, 2024). Among the practicing nurses, the immigrant nursing workforce constitutes 33 % (26,887) of the total number in Ireland (NMBI, 2022). Within the migrant nursing workforce, 90 % of new nurses are arriving in Ireland from India yearly (NMBI, 2023). In 2023, the top overseas countries from which new registrants nurses came were India (3272), Philippines (560), United Kingdom (232) and Zimbabwe (169) (NMBI, 2023).

Work-related stress is a universal phenomenon that has been found to contribute to adverse health effects, impaired performance, and general well-being concerns (Akanji, 2013). Occupational stress, or job stress, occurs when there are discrepancies between the physiological demands within a workplace and employees' ability to manage or cope with such demands (Akanji, 2013). HCWs may encounter work-related stress (Sadath and Kumar, 2017) due to organizational issues, disparities in demands, inadequacy of skills, lack of workplace social support systems, or a combination of these factors (Ruotsalainen et al., 2015). HCWs, particularly nurses, face an increasingly challenging task of delivering the highest standard of care while contending with insufficient resources (Farsi et al., 2010; OECD, 2015). Nurses often experience high burnout (Chigwedere et al., 2021), which is associated with factors such as high workload, value incongruence, low control over the job, limited decision latitude, poor social climate/social support, and low rewards (Dall'Ora et al., 2020). Furthermore, shift work is indispensable in healthcare, but it can have detrimental effects on the physical, mental, and social well-being of the workers (Kim et al., 2016).

Migration is the act of relocating from one cultural environment to another, intending to settle either temporarily or permanently (Schouler-Ocak et al., 2020). Migrant workers undergo acclimatization of the sociocultural conditions of the host country, which may differ from those in their home countries (Liem et al., 2021). They face multiple challenges related to cultural practices, religious beliefs, language barriers, discrimination, and racism (Alarcon, 2022).

A recent systematic review, involving 44,365 migrant workers in 17 different countries (although not specific to the HCWs), indicates a high prevalence of depression (39 %) and anxiety symptoms (27 %) among this group (Hasan et al., 2021). Job factors such as workplace psychosocial stressors, poor working conditions, salary and benefits issues, abuse, and limited social support available were associated with mental health outcomes in migrant workers (Hasan et al., 2021). Another systematic review of 14 empirical studies conducted among migrant and minority nurses concludes that migrant nurses are at a higher risk of work-related injuries and discrimination than native or majority ethnicity nurse(Schilgen et al., 2017).

Studies addressing the mental health of migrant HCWs are inconclusive. For instance, overseas employees in Sweden reported higher levels of mental discomfort, whereas expatriate workers in China exhibit better mental health (Dunlavy and Rostila, 2013; Li et al., 2014). Considering these heterogeneous results, it is essential to generate context-specific knowledge on migrant health and mental health across host countries. Understanding the type of stressors experienced by migrant HCWs would encourage policymakers in the healthcare system to create and implement programs aimed at enhancing their quality of life (Connor and Miller, 2014). To the best of our knowledge, little or no evidence exists on migrant HCWs' mental health in Ireland.

Therefore, we embarked on examining: 1) The level of occupational stress and psychological distress experienced by migrant HCWs in Ireland 2) Is there a difference in the experience of occupational stress and psychological distress among nurses and Heath Care Assistants (HCAs) ? 3)What demographic and occupational-related factors are associated with job stress and psychological distress among this group? Additionally, as a secondary objective, we aim to examine the Job stress and psychological distress among migrant HCWs of different nationalities.

## Materials and methods

The study received ethical approval from the University College Cork (UCC), School of Public Health Social Research Ethics Committee. Explicit consent was obtained online using the University College Cork (UCC) approved Google form from all the study participants.

A cross-sectional online survey using Google Forms was conducted among overseas nursing and HCAs currently employed (full-time/part-time) in the Republic of Ireland. The study included individuals who met the following criteria: a) currently practicing overseas nurses and HCAs in Ireland, b) overseas nurses and HCAs who had been working in Ireland for a minimum of 6 months, and c) healthcare workers who were at least 18 years of age. Potential healthcare workers for this study were identified via social media platforms, including WhatsApp groups such as "Migrant HCWs in Ireland," which has approximately 1000 members, and a WhatsApp group of healthcare workers in a large private hospital in Dublin, which has 250 members. The Google Forms were sent to these WhatsApp groups with a weekly reminder message requesting their participation in the survey. Data collection for this study took place between April 20, 2023, and May 20, 2023. The study employed a nonprobability consecutive sampling method for data collection. Participation was voluntary and anonymous. Only those who electronically consented to the study had access to the questionnaire.

### Measures

Minimal information on participants' demographic and job characteristics, such as gender, age range, area of work, years of experience, employment details, country of origin, was collected using a self-prepared checklist.

Occupational stress and psychological distress were measured using the Brief Job Stress Questionnaire (BJSQ)(Shimomitsu, 2000) and the Kessler Psychological Distress Scale (K6)(Kessler et al., 2003).

### Occupational stress

The BJSQ (Shimomitsu, 2000) was employed to assess the work-related stress of the study participants. The BJSQ has 57 items comprises of 3 factors: Job stressors (17 items), Stress responses (29 items), 3) Social support and related factors (9+2 items). The BJSQ uses a 4-point Likert scale (1 = strongly disagree to 4 = strongly agree) with a reverse score for Items 1–7 and 11–13 for Job Stressors and items 1–3 for Stress Responses. A high score denotes higher stress on the scales. However, a higher score on social support and related items indicated lower level of social support and or satisfaction.

The Job Stressors (17 items) are sub classified into quantitative overload (items 1– 3), mental demand (items 4–6), physical workload (item 7), job control (items 8–10), utilization of techniques (item 11), interpersonal relationships (items 12–14), work environment (item 15), fit to the job (item 16), and reward for the work (item 17). The Stress Responses (29 items) placed into subscales as: lack of vigor (items 1–3), irritability (items 4–6), fatigue (items 7–9), anxiety (items 10–12), depression (items 13–18), and somatic symptoms (items 19–27). Finally, 11 items of the Social Factors are sub-categorised as supervisor support (items 1, 4, and 7), co-worker support (items 2, 5, and 8), support from spouse, family, and friends (items 3, 6, and 9), and job satisfaction (items 10 and 11)(Shimomitsu, 2000).

### Psychological distress

Mental health of the participants was assessed by utilising Kessler Psychological Distress Scale (k6)(Kessler et al., 2003). This scale consists of six items designed to assess the emotional state of an individual, including feelings of nervousness, hopelessness, restlessness, depression, and sadness experienced within the last 30 days with five possible responses (ranging from 0=none of the time to 4=all of the time). A total score (range: 0–24) is generated by adding the item scores. A score of 13 or higher on the scale indicates serious psychological distress(Kessler et al., 2003).

### Statistical analysis

The quantitative data collected through an online survey were exported from Google Forms to Excel for data cleaning and then transported into SPSS. Data analysis was performed using SPSS® (Version 27). Descriptive analysis summarized frequencies and the distribution of participants' demographic variables, including mean and standard deviation (SD) of the BJSQ and K6 scale items. The internal consistency of the scale was measured using Cronbach's Alpha(Cronbach, 1951). Cronbach's Alpha indicated a value of 0.942 for the BJSQ scale and 0.923 for the K6 scale, demonstrating good internal reliability and indicating that the items in both scales accurately measure a similar concept. Additionally, the BJSQ total scale score and K6 scale score were normally distributed (Shapiro– Wilk test = p > 0.05).

To further explore sub-group analysis between nurses and HCWs, an independent sample *t*-test was employed. One-way ANOVA was performed to examine if there are differences in job stress and psychological distress among the participants across nationalities, and simultaneously accounting for country of birth.

Multivariable linear regression models were conducted to examine the predictors of occupational stress and psychological distress among the participants. Two regression models were performed, treating occupational stress (overall score combining of the three major subscales) as the dependent/outcome variable in model 1, and psychological distress as the dependent/outcome variable in model 2. The predictors included in the models were gender (female=1; male=0), job title (nurse=1; HCA=0), years of experience (<10 years=1; 10 years or more=0), place of work (Private sector=1; Health Service Executive or other public sector=0), and the current role of the nurse (staff nurse=1; nurse manager or similar role=0). The same set of predictors was entered into both models.

## Results

### Demographic profile of the participants

The response rate was 35.8 % (*n* = 447) from the 1250 invitees. The respondents were largely nurses (*n* = 416; 93.1 %), while the remaining 31 (6.9 %) were HCAs. Females accounted for 363 respondents (81.2 %), and 83 (18.6 %) were males., The largest proportion of respondents were from India 325 (72.7 %), followed by 79 (17.7 %) from the Philippines, 26 (5.8 %) represented Africa, and 6 (1.3 %) from EU countries except Ireland. The remaining 11 (2.5 %) participants were from other parts of the world. A total of 209 participants belonged to the age group of 35–44. Additionally, 127 participants were between the ages of 25 and 34, while 87 individuals represented the 45–54 age group. The remaining 30 participants were aged 55 and older. See Table 1 for more details.

**Table 1:** Sociodemographic profile of the study participants.

Job Title	Frequency (%)	Area of speciality	Frequency (%)
Nurse	416 (93.1)	Surgical	90 (20.1)
Health Care assistant	31 (6.9)	other	71 (15.9)
Gender		Medical	63 (14.1)
Female	363 (81.2)	care of elderly	60 (13.4)
Male	83 (18.6)	Operating theatre	37 (8.3)
Other	1 (0.2)	Critical care	36 (8.1)
Age		oncology	20 (4.5)
25–34	127 (28.4)	Midwifery	19 (4.3)
35–44	209 (46.8)	Emergency Department	18 (4)
45–54	87 (19.5)	intellectual disability	15 (3.4)
55–64	21 (4.7)	Paediatrics	10 (2.2)
65 and above	3 (0.7)	Psychiatry	8 (1.8)
Marital status		Current working organisation	
Married	385 (86.1)	Private Hospital	200 (44.7)
Single	38 (8.5)	HSE Hospital	175 (39.1)
In a relationship	9 (2)	Other Health care facility	56 (12.5)
separated	5 (1.1)	HSE owned nursing Home	16 (3.6)
widowed	4 (0.9)	Working status	
prefer not to say	4 (0.9)	Full time permanent	408 (91.3)
other	2 (0.4)	Part time permanent	28 (6.3)
current Role		Full time temporary	8 (1.8)
Staff Nurse	314 (70.2)	Other	3 (0.7)
Clinical Nurse Manager/ Specialist	82 (18.4)	Country of origin	
Clinical Nurse Manager-3	4 (0.9)	India	325 (72.7)
Assistant Director of nursing	10 (2.2)	Philippines	79 (17.7)
Director of nursing	4 (0.9)	Africa	26 (5.8)
Advanced Nurse Practioner (ANP)	2 (0.4)	Other	11 (2.5)
Health Care Assistant	31 (7)	Europe except Ireland	6 (1.3)
Working period in Ireland			
10 years and above	179 (40)		
6 months to 3 years	157 (35.1)		
3–6 years	83 (18.6)		
6–9 years	28 (6.3)		

### Descriptive results of the BJSQ and K6 scales

Table 2 presents the descriptive results of the BJSQ and K6 scale domains. The average score for the job stressors factor was 44.9 (SD 6.8; range 25 to 66), indicating medium to high job stress. The stress response factor showed an average of 57.1 (SD 14.4; range 30 to 115), implying a low stress response. While a higher score on social support and related items indicated lower social support, the current score was 20.70 (SD 5.4; range 11 to 35), indicating poor social support for the participants.

**Table 2:** Descriptive results of the BJSQ and K6 scales.

Items	Mean (SD)	Range
BJSQ major factors/domains		
Job Stressors	44.9 (6.8)	25–66
Stress response	57.1 (14.4)	30–115
Social Factors	20.7 (5.4)	11–35
Job Stressors		
Quantitative work overload	8.7 (5.1)	3–12
Mental demand	10.4 (1.8)	3- 12
Job Control	7.7 (2.3)	3–12
Interpersonal relationships	6.1 (2.1)	3–12
Physical workload	3.1 (1.0)	1–4
My knowledge and skills are rarely used at work	2.1 (1.1)	1–4
My working environment is poor (e.g. noise, lighting, temperature, ventilation)	3.2 (1.0)	1–4
This job suits me well	1.8 (1.0)	1–4
Stress response		
Lack of vigor	5.9 (2.5)	3–12
Irritability	6.1 (1.7)	3–12
Fatigue	7.0 (2.1)	3 −12
Anxiety	6.2 (2.0)	3 - 12
Depression	12.3 (3.9)	7 −28
Somatic symptoms	17.5 (5.2)	9 - 36
Social support		
support from superiors	6.8 (2.3)	3 −12
support from Co worker	6.50(1.9)	3 - 12
Support from Spouse Family & Friends	4.3(1.7)	3 −10
K6 total score	7.2 (5.4)	0 −24

Note: A higher score on social support items indicates a lower level of social support.

Analysis of the sub-domains of job stressors indicated that participants reported high stress on quantitative overloads with a mean score of 8.7 (SD 5.1; range 3 to 12) and mental demands at work with an average score of 10.45 (SD 1.8; range 3 to 12). However, they reported a medium level of stress on job control with a mean score of 7.7 (SD 2.3; range 3 to 12). The stress score of the interpersonal relationships factor had a mean score of 6.1 (SD 2.1; range 3 to 12), indicating a low to medium level of stress. The item assessing physical workload, ‘My job requires a lot of physical work,’ had an average score of 3.08 (SD 0.9; range 1 to 4), reflecting a high physical workload.

Regarding the -domains of stress response, irritability (mean 6.1; SD 1.7; range 3 to 12), fatigue (mean 7.0; SD 2.2; range 3 to 12), anxiety (mean 6.2; SD 2.1; range 3 to 12), somatic symptoms (mean 17.5; SD 5.3; range 9 to 36), depression (mean 12.3; SD 3.92; range 7 to 28), and lack of vigor (mean 5.9; SD 2.5; range 3 to 12) were expressed as a low level by the participants.

While the participants perceived poor social support overall, more issues on social support were found in the domains of support from superiors and co-workers. However, the participants perceived higher support from family and friends.

Analysis of the K6 scale revealed a low level of psychological distress among the respondents with an average score of 7.2 (SD 5.4; range 0 to 24).

### Comparison of occupational stress and psychological distress between nurses and healthcare assistants: an independent sample *t*-test analysis

Table 3 presents an independent sample *t*-test, which was conducted to compare job stress and psychological distress among nurses and HCAs. The analysis shows no statistically significant differences in job stress (*t* = −1.573; *p* = 0.116) or stress response (*t* = −0.83; *p* = 0.407) between the nurses and HCAs. However, there was a statistically significant difference in social support (*t* = −2.549; *p* = 0.011) and psychological distress (*t* = −1.991; *p* = 0.047) where HCAs experienced more issues in social support or a lower level of social support and high psychological distress as compared to nurses.

**Table 3:** Comparison of occupational stress and psychological distress between nurses and healthcare assistants: an independent sample *t*-test analysis.

Variables	Mean (SD)	t-value	p- value
Job stress			
Nurse	44.7 (6.9)	−1.573	0.116
HCAs	46.7 (6.6)		
Stress responses			
Nurse	56.9 (14.5)	−0.83	0.407
HCAs	59.2 (13.6)		
Social support			
Nurse	20.5 (5.3)	−2.549	0.011
HCAs	23.1 (6.3)		
Psychological distress (K6 scale)			
Nurse	7.1 (5.4)	−1.991	0.047
HCAs		9.1 (5.4)	

Note: A higher score on social support items indicates a lower level of social support.

### Predictors of job stress and psychological distress

Table 4 shows the predictors of job stress among the participants, using multiple linear regression models. The F-test indicated an overall model fit at *p* = 0.001, *F* = 7.074. Migrant nurses and HCAs who have worked in Ireland for <10 years perceived higher job stress (*t* = 2.826; β = 0.154; *p* = 0.001), whereas working in private healthcare sectors was associated with a lower perception of job stress (*t* = −3.077; β = 0.15; *p* = 0.001). The model explains **24.4 %** of the variation in the dependent variable (Adjusted R^2^= 0.244).

**Table 4:** Predictors of job stress among the migrant HCWs.

Model-1		Unstandardized Coefficients	Standardized Coefficients		
	B	Robust Std. Error^a^	Beta	t	Sig.
(Constant)	127.292	4.743		26.839	.000
Gender (female=1; male=0)	1.990	2.840	.034	.701	.510
Nurse (nurse=1; HCAs=0)	−8.233	4.716	−0.091	−1.746	.077
<10 years (<10 years=1; >10 years)	7.225	2.557	.154	2.826	.001
Private sector (private sector=1; public sector=0)	−6.934	2.254	−0.150	−3.077	.001
Staff nurse (staff nurse=1; =nurse supervisor=0)	1.614	2.816	.032	.573	.506

Dependent Variable: Overall job stress; Adjusted R^2^= 0.244.

Model 2 examined the predictors of psychological distress using the same independent variables applied in Model 1 (Table 5). The F-test indicated an overall model fit at *p* < 0.001, *F* = 8.211. Consistent with Model 1, those who have worked for <10 years in Ireland (*t* = 5.666; β = 0.303; *p* = 0.000) perceived higher psychological distress, while working in private healthcare sectors was associated with less psychological distress (*t* = −2.643; β = 0.126; *p* = 0.008). However, other predictors in the models, such as gender, nurse vs. HCAs, and staff nurse vs. nurse supervisor role, were not associated with either job stress or psychological distress. The model explains 28.2 % of the variation in the dependent variable (Adjusted R^2^= 0.288).

**Table 5:** Predictors of psychological distress among the migrant HCWs.

	Unstandardized Coefficients		Standardized Coefficients		
Model-1	B	Robust Std. Error^a^	Beta	t	Sig.
(Constant)	7.182	1.122		6.598	.000
Gender (female=1; male=0)	.995	.656	.072	1.527	.130
Nurse (nurse=1; HCAs=0)	−1.540	1.161	−0.073	−1.423	.185
<10 years (<10 years=1; >10 years)	3.325	.567	.303	5.666	.000
Private sector (private sector=1; public sector=0)	−1.367	.510	−0.126	−2.643	.008
Staff nurse (staff nurse=1; =nurse supervisor=0)	−0.796	.644	−0.068	−1.232	.217

Dependent Variable: Psychological distress; Adjusted R2= 0.288.

### Job stress and psychological distress among migrant HCWs with different nationalities

The results of the one-way ANOVA showed no statistically significant differences in both job stress (*F* = 1.230; *p* = 0.297) and psychological distress (*F* = 0.319; *p* = 0.865) among migrant HCWs with various nationalities, such as Indian, Filipino, African, or European, excluding Ireland.

## Discussion

The present study examined the extent of occupational stress and psychological distress experienced by migrant HCWs in Ireland, as well as the differences in these variables among migrant nurses and HCAs, and the demographic and occupational-related factors associated with job stress and psychological distress among HCWs. The results of the study indicated that HCWs experienced moderate to high job stress and moderate levels of psychological distress, with significant differences between nurses and HCAs in terms of social support and psychological distress. Having <10 years of work experience in Ireland was identified as a significant risk factor while working in the private healthcare field was associated with a lower risk of occupational stress and psychological distress.

The descriptive results indicated moderate to high job stress, with a relatively high-stress score on quantitative overloads, mental demands at work, and physical work demands. Additionally, they indicated poor social support among migrant HCWs. However, the current study's findings indicated low levels of stress responses. Previous studies have reported a moderate level of stress experience (Jiaru et al., 2023), emotional exhaustion, work overload, and work-related stress among nurses (López-López et al., 2019). Increasingly high workloads, poor working conditions, lack of recognition, and high levels of occupational stress are reported among healthcare support workers in residential homes (Czuba et al., 2019). The nurses' workplace, in general, is characterized by physically and psychologically demanding tasks, including shift work, while that of migrant nurses is additionally characterized by discriminatory practices (Schilgen et al., 2017) and high level of acculturation stress (Adebayo et al., 2021). While it is encouraging to observe the low level of stress response, specifically low levels of irritability, anxiety, somatic, and depressive symptoms among migrant HCWs in our study, it is worrying that they have perceived high stress and poor social support, which are poor mental health indicators (Koelmel et al., 2017). Limited social support was associated with poor mental health outcomes in migrant workers (Hasan et al., 2021; Koelmel et al., 2017). It is also concerning to observe that HCWs perceived poor social support, specifically from colleagues and supervisors.

While some aspects of psychological distress are being captured by the stress response subscale of BJSQ, the K6 scale measures served a complementary role. The current findings on K6, indicating that HCWs experience a moderate level of psychological distress, are quite similar to the findings on the stress response subscale. However, the finding is not in agreement with a previous study among migrant nurses in Germany, which reported high psychological distress among both migrant and non-migrant nurses, and significantly high depressive and somatic symptoms among migrant nurses(Schilgen et al., 2020). Nonetheless, there are very few studies available on occupational stress and the mental health of migrant healthcare workers.

We found no significant differences in job stress or stress response between nurses and HCAs. However, statistically significant differences were found in social support and psychological distress, with HCAs reporting lower levels of social support and higher psychological distress compared to nurses. Notably, healthcare support workers have consistently reported poor working conditions, insufficient recognition, and elevated levels of occupational stress (Czuba et al., 2019). Although there haven't been prior comparative studies specifically investigating the social support or psychological distress of migrant nurses versus HCAs, a study from Australia did highlight significantly high acculturation stress among migrant nurses in residential care facilities compared to care assistant role(Adebayo et al., 2021). It is crucial to note that our focus was on occupational stress and psychological distress rather than acculturation stress, and we consider the current findings to be exploratory in nature.

The years of work experience in the host country were a significant predictor of both job stress and psychological distress, and those with 10 years or less of work experience had experienced higher job stress and psychological distress. Potential explanations are: Firstly, the experience contributes to an increase in job skills and capacities to perform the required roles as a nurse or HCA, potentially resulting in less job stress or distress(Xiao et al., 2014). Secondly, long years of work experience in the host country also indicate the length of residence in the host country, and therefore, better adaptability and integration into the culture of the host country, resulting in a better support system and reduced acculturative stress (Martinovic et al., 2009). The initial years of employment are the most difficult period for migrant nurses as they experience multiple challenges in dealing with cultural shock, conflict resolution, and intercultural communication while adapting their nursing knowledge and practice to the host culture(Xiao et al., 2014). Lack of experience and expertise was a major barrier for the nurses to integration with the host work environment (Xiao et al., 2014).

Interestingly, we have identified significantly elevated levels of job stress and psychological distress among HCWs employed in the public healthcare sector compared to those in the private sector. A recent prospective study from Sweden reported that nursing professionals working in the public healthcare sector experienced higher job demands and lower social support compared to their counterparts in the private healthcare sector(Thapa et al., 2022). Other studies also documented increased job stress among nurses in the public sector(Bojčeski et al., 2019) compared to the private sector(Tsegaw et al., 2022).

However, there is limited evidence on psychological distress or job stress among HCWs working in the public versus private healthcare sector, and we believe that the local context and situation could play a crucial role in determining these variables. Additionally, occupational stress and psychological distress may be influenced by various factors, which we have not included in our regression models. For instance, the area of specialty could be one such factor among many others. A previous study from Ireland reported significantly high occupational stress among nurses working in medical wards, accident and emergency, intensive care units, and pediatric wards (McCarthy et al., 2010). Although our data include the area of specialty, we have not incorporated it into the models due to the exhaustive number of specialties and considering the sample size. Additionally, factors such as lack of resources, staff shortages, poor working conditions[38], increased workload, low pay rates, and challenging relationships with colleagues and managers can contribute to heightened job stress(Ramon, 2021).

The study implications point to offering better and tailored stress management programs to immigrant nurses and HCAs; initiating strategies to improve cohesion and support, including mentoring programs. Such programs can assist first-generation immigrant nurses in minimizing the impact of challenges they face during their adaptation to the new nursing workforce (Ham, 2021). Additionally, regional nurses and nursing managers must be prepared to recognize diversity in the nursing profession and adjust accordingly to cater to the unique requirements of immigrant nurses(Buttigieg et al., 2018).

### Strengths and limitations

The study has several strengths. To the best of our knowledge, it provides first-hand evidence on migrant HCWs' mental health in Ireland, which is relevant and timely given the recent increase in migrant HCWs in the country. Addressing their occupational stress and psychological distress is crucial not only for their own well-being but also for ensuring patient safety. Given the scarcity of literature in this area, the current findings will contribute context-specific knowledge on migrant HCWs' mental health. The current findings also imply the need for specific workplace mental health intervention studies focusing on migrant HCWs.

A limitation of the study lies in the fact that the sample predominantly comprises a selective population, which may not be representative of all healthcare facilities in Ireland. The study may be underpowered. However, the response rate is acceptable given the recent phenomenon of 'survey fatigue'. Another limitation of this study is the underrepresentation of healthcare assistants (HCAs) in the sample, which may impact the generalizability of findings regarding this group. While the analysis did not find statistically significant differences in job stress between nurses and HCAs, HCAs reported lower social support and higher psychological distress. The small sample size of HCAs (6.9 %) might have reduced statistical power to detect meaningful differences. Future studies should aim to increase HCA participation and replicate the analysis with a more balanced representation of healthcare worker groups to strengthen conclusions about occupational stress and mental health among HCAs. This is a cross-sectional study, so direct causal inference is not possible. The current study findings are exploratory in nature, and more studies, particularly prospective studies, are needed for a reliable conclusion.

## Conclusion

This study examined occupational stress and psychological distress among migrant HCWs in Ireland, compared experiences between nurses and healthcare assistants, and identified key demographic and occupational predictors. Findings revealed that migrant HCWs experienced moderate to high job stress and moderate psychological distress, with less experienced HCWs (under 10 years) being at higher risk. Working in the private sector was linked to lower stress and distress, while gender, job role, and position were not significant predictors. No significant differences were found across nationalities.

Job experience and workplace setting play a crucial role in migrant HCWs' mental health. Addressing occupational stress requires policies that consider these workplace factors to improve well-being and retention. Future research should explore the long-term mental health trajectories of migrant HCWs in Ireland.

## CRediT authorship contribution statement

**Sindhu Thankachen:** Writing – review & editing, Writing – original draft, Visualization, Validation, Software, Resources, Investigation, Formal analysis, Data curation, Conceptualization. **Zubair Kabir:** Writing – review & editing, Supervision, Methodology, Conceptualization. **Anvar Sadath:** Writing – review & editing, Writing – original draft, Visualization, Validation, Supervision, Software, Resources, Project administration, Methodology, Investigation, Formal analysis, Data curation, Conceptualization.

## Declaration of competing interest

The authors declare that they have no known competing financial interests or personal relationships that could have appeared to influence the work reported in this paper.
